# Utilization of BiLSTM- and GAN-Based Deep Neural Networks for Automated Power Amplifier Optimization over X-Parameters

**DOI:** 10.3390/s25175524

**Published:** 2025-09-05

**Authors:** Lida Kouhalvandi

**Affiliations:** Department of Electrical and Electronics Engineering, Dogus University, 34775 Istanbul, Türkiye; lida.kouhalvandi@ieee.org

**Keywords:** automated, bidirectional long short-term memory (BiLSTM), deep neural network (DNN), generative adversarial network (GAN), multi-objective optimization, power amplifier (PA), X-parameters

## Abstract

This work proposes a design technique to facilitate the design and optimization of a highperformance power amplifier (PA) in an automated manner. The proposed optimizationoriented strategy consists of the implementation of four deep neural networks (DNNs), sequentially. Firstly, a bidirectional long short-term memory (BiLSTM)-based DNN is trained based on the X-parameters for which the hyperparameters are optimized through the multi-objective ant lion optimizer (MOALO) algorithm. This step is significant since it conforms to the hidden-layer construction of DNNs that will be trained in the following steps. Afterward, a generative adversarial network (GAN) is employed for forecasting the load–pull contours on the Smith chart, such as gate and drain impedances that are employed for the topology construction of the PA. In the third phase, the classification the BiLSTM-based DNN is trained for the employed high-electron-mobility transistor (HEMT), leading to the selection of the optimal configuration of the PA. Finally, a regression BiLSTMbased DNN is executed, leading to optimizing the PA in terms of power gain, efficiency, and output power by predicting the optimal design parameters. The proposed method is fully automated and leads to generating a valid PA configuration for the determined transistor model with much more precision in comparison with long short-term memory (LSTM)-based networks. To validate the effectiveness of the proposed method, it is employed for designing and optimizing a PA operating from 1.8 GHz up to 2.2 GHz at 40 dBm output power.

## 1. Introduction

Power amplifiers (PAs) are promising solutions in fifth-generation (5G) systems that amplify related signals before transmission [1] and provide solutions for generating wide bandwidths [2]. Hence, high performance and suitable outcomes are required to tackle the emerging drawbacks. Recently, the execution of various kinds of neural networks (NNs) has proven its effectiveness in designing and optimizing radio frequency (RF) designs [3,4] for various purposes.

In [5], an artificial neural network (ANN) is employed for estimating the nonlinear characteristics of transistors over distributed frequencies. In other studies ([6,7]), ANNs are used for Digital predistortion (DPD), which is significant for PA linearization. As another type of ANN, in [8], a recurrent neural network (RNN) is constructed for DPD linearization along with PA behavioral modeling. In [9], a multigroup aggregation neural network (MGANN) is employed for accurately modeling a PA based on the product term of I/Q components. A convolutional neural network (CNN) is used in [10] for estimating the S-parameters of electromagnetic (EM) layouts. Also in [11], an augmented CNN is used for linearizing PA performance. An augmented real-valued time-delay neural network is used in [12] to enhance baseband intermodulation distortion in PAs. A deep neural network (DNN) is used for PA modeling in [13] for minimizing the number of chosen input terms [14,15].

A convolutional long short-term deep neural network (MCLDNN) approach is employed in [16] for modeling a nonlinear PA design, along with decreasing the complexity of networks. In another study, [17], an ANN-based DPD technique is used, leading to the presentation of equivalent linearization performance with the help of terahertz waves. Additionally, in [18], a learnable edge-located activation neural network is used for improving the flexibility of nonlinear modeling, along with reducing the complexity. A graph neural network (GNN) is used in [19] for enhancing the overall performance of cell-free massive multiple-input multiple-output (CF-mMIMO) systems, which include nonlinear PAs. In [20], an ANN-based method is employed for accurate large-signal modeling operating near the threshold region. A DNN is executed in [21], which synthesizes the layout of matching networks used in PAs.

This work is devoted to presenting an automated optimization process in which two different DNN structures, BiLSTM and GAN, are used for enhancing the overall performance of a PA. Firstly, the regression bidirectional long short-term memory (BiLSTM)-based DNN is trained for modeling the high-electron-mobility transistor (HEMT) through X-parameters. This phase is essential for determining the hyperparameters of the network through multi-objective optimization methods. Here, various multi-objective optimizations are employed and are compared with the combination of long short-term memory (LSTM) and BiLSTM DNNs to prove the effectiveness of the BiLSTM topology. Afterward, a GAN is trained to achieve the optimal gate and drain impedances of the HEMT transistor, which are necessary for constructing the initial structures of input and output matching networks (MNs). As the third phase of optimization, another BiLSTM-based DNN is constructed for predicting the most suitable PA structure for the determined outcomes. And as the last phase, the regression BiLSTM-based DNN is employed for sizing the PA. This methodology is fully automated, in which the hyperparameters of the trained network are predicted through X-parameters, and after that, with the help of this information, classification and regression BiLSTM-based DNNs are constructed for generating the PA structure, along with sizing it, respectively. The GAN also plays an important role, since with the predicted impedances, the optimal structure of the PA is generated. The proposed method is validated by designing and optimizing a high-performance PA with the help of an Ampleon CLF1G0060-10 Gallium Nitride (GaN) HEMT operating with a bandwidth of 400 MHz.

This paper is organized as follows: Section 2 presents an intelligence-based methodology leading to modeling and optimizing the high-performance PA through various DNNs. Section 3 explains the practical implementation of the proposed approach. Section 4 is devoted to providing the simulation results of the optimized PA, and finally, Section 5 concludes this work.

## 2. Proposed Optimization Method Based on DNNs

**Figure 1:**
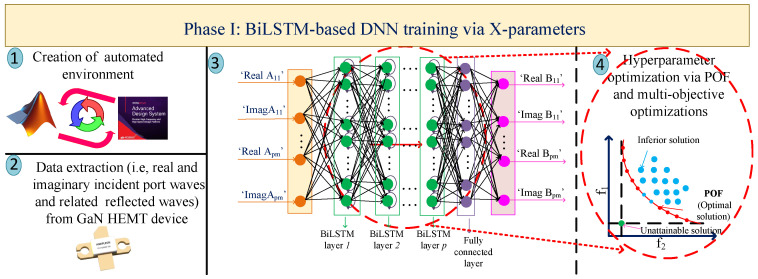
Training BiLSTM-based DNN through X-parameters for determining optimal hyperparameters.

**Figure 2:**
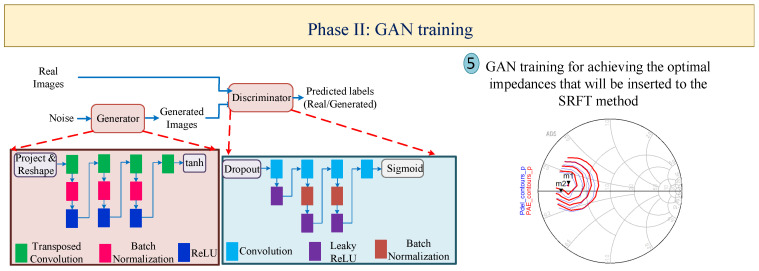
GAN construction for predicting the optimal gate and drain impedances of the employed HEMT device.

**Figure 3:**
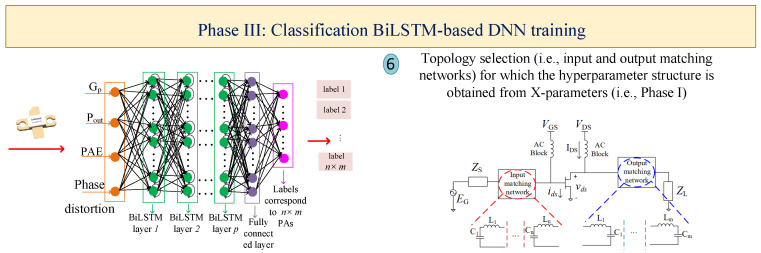
Classification BiLSTM-based DNN for predicting the most suitable MNs for configuring the PA.

**Figure 4:**
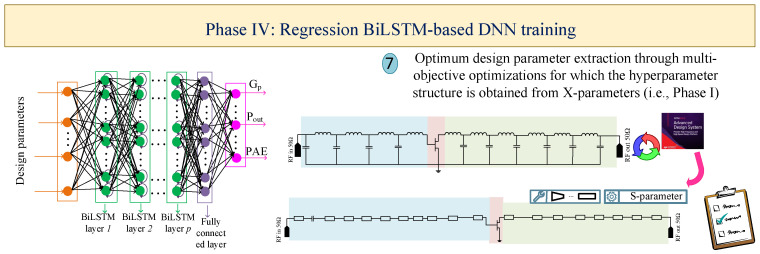
Regression BiLSTM-based DNN for obtaining the optiaml design parameters.

Designing and optimizing nonlinear circuits, such as PAs, requires advanced methodologies and approaches that are time-consuming and depend on the experience of designers. To tackle this problem, this section presents the automated methodology based on the utilization of two types of ANNs, BiLSTM and GAN, leading to the following: (I) firstly constructing a high-accuracy DNN with the help of X-parameters; (II) determining the optimal impedances for generating the configuration of MNs through the GAN; (III) estimating the optimal topology of the PA through the classification BiLSTM network for which the hidden-layer structure is determined from the constructed DNN via X-parameters; (IV) predicting the optimal design parameters through the regression BiLSTM DNN, leading to having high performance outcomes that the DNN structure generates from the first phase. The general flowcharts for the proposed four phases are depicted in Figure 1, Figure 2, Figure 3 and Figure 4, respectively. For the BiLSTM-based networks, the rectified linear unit (ReLU) function is employed as the activation function, and the normalized root mean square error (RMSE) is used for determining the convergence factor. Additionally, at the end of this section, in detail, the steps for the presented four phases are summarized in Algorithm 1.
**Algorithm 1** Proposed automated methodology based on BiLSTM-based DNNs and GAN for modeling and sizing PA**1**: Combination of EDA tool (here, Keysight ADS) with numerical analyzer (here, Matlab) for co-design simulations;**2**: Extraction of incident port waves through independent TCAD physical simulator leading to provide the X-parameter data of employed HEMT device;**3**: Training the regression BiLSTM-based DNN through X-parameters;**4**: Implementation of multi-objective optimizations leading to predict the optimal hyperparameters of network;**5**: GAN network training for predicting the optimal gate and drain impedances of employed HEMT device that these impedances will be inserted to the SRFT method for generating MNs of PA;**6**: Construction of classification BiLSTM-based DNN for predicting the optimal PA configuration that is modeled through the achieved optimal impedances in the previous step;**7**: Training the regression BiLSTM-based DNN for estimating the optimal design parameters leading to obtain the targeted specifications.

### 2.1. Phase I: BiLSTM-Based DNN Construction with the Help of X-Parameters

In constructing and training any DNN, determining the optimal hyperparameters of the network, including the number of hidden layers with neurons, is significant and requires careful efforts [22]. Hence, as the first step, an intelligence-based method for obtaining the hyperparameters is presented. In our proposed method, the automated environment is created with the combination of an electronic design automation (EDA) tool (here, Keysight ADS, 2024) and numerical analyzer (here, MATLAB, 2024) (Step-1). Afterwards, the real and imaginary incident port waves $A_{k,l}$ (where *k* is the port index and *l* the harmonic index) along with the corresponding reflected waves are extracted from an independent TCAD physical simulator [23] for the determined GaN HEMT device (Step-2). More details regarding the extraction of X-parameters are presented in [24]. By creating an automated environment and obtaining the necessary data for training the network, a BiLSTM-based DNN, depicted as the 3rd step (Step-3) in Figure 1, is constructed. As shown, the input layer consists of the information of incident port waves, and the output layer presents the related reflected waves as responses for each data point of the input layer. The random incident waves $A_{11}$, $A_{12}$,..., $A_{1n}$ with the corresponding reflected waves $B_{11}$, $B_{12}$,..., $B_{1n}$ are extracted and three types of data, training ($X_{\mathrm{Train}}$), validation ($X_{\mathrm{Val}}$), and testing ($X_{\mathrm{Test}}$) data, along with $Y_{\mathrm{Train}}$, $Y_{\mathrm{Val}}$, and $Y_{\mathrm{Test}}$, which are the corresponding data of the input data, are arranged for training the network. With these arrangements, the DNN network is trained and the hyperparameters of this network are optimized through the implementation of multi-objective optimizations that are based on the Pareto optimal front (POF) [25,26,27] (Step-4).

### 2.2. Phase II: GAN Training for Obtaining the Optimal Impedances of the HEMT Device

The simplified real frequency technique (SRFT) is an effective method in generating input and output MNs [28] for the inserted gate and drain impedances of transistors. As Equation (1) presents, the generation of transducer power gain depends on the matrix $h_{n}$ for all *n* ≥ 3; hence, various numbers of MNs can be generated. With the generated various MNs, the question that emerges is what is the optimal MN structure for the executed HEMT device? For this case, selecting the optimal impedances along with the MNs is critical. This section (Phase II) is devoted to presenting the methodology for selecting the optimal gate and drain impedances that will be inserted into the SRFT method, and the next phase (i.e., Phase III, presented in the next section) will explain the approach for predicting the most suitable MNs generated from the SRFT method.

(1) $$G_{n}={\left(-1\right)}^{n}h_{n}^{2}.$$

As Figure 2 presents, the GAN is trained for predicting the optimal gate and drain impedances of the employed HEMT device (Step-5). As is obvious, the GAN includes ’generator’ and ’discriminator’ sections in which these parts operate against each other until the generator produces the realistic data [29]. Here, the generator network is built for generating the load–pull contours along with the load–pull images, and the discriminator network learns with respect to the valid load–pull contours.

### 2.3. Phase III: Classification BiLSTM-Based DNN for Obtaining the Optimal Configuration

After obtaining the optimal gate and drain impedances through the GAN, with the help of the presented methodology in Figure 3, a classification BiLSTM-based DNN is targeted to be trained (Step-6). Here, the SRFT method is employed for obtaining the various generated MNs for input and output parts [28]. With the various generated structures, the important question that emerges is what is the optimal and suitable PA structure? To tackle this problem, the nominated DNN is constructed in which the input-layer specifications are power gain ($G_{p}$), output power ($P_{out}$), power-added efficiency (PAE), and phase distortion. The output layer represents the class number for the determined PA structure.

With the help of hyperparameters achieved in Phase I, along with the dataset obtained by randomly iterating the geometrical values of various PA structures [30], the DNN of this section is trained. After constructing the network, the specifications of the utilized HEMT transistor are inserted into the network, and the DNN predicts the most suitable PA topology that fits the targeted outcomes.

### 2.4. Phase IV: Regression BiLSTM-Based DNN for Obtaining the Optimal Geometric Parameters

As the last step of the proposed methodology, another BiLSTM-based DNN is trained for achieving the optimal design parameters (i.e., geometric values) (Step-7). As shown in Figure 4, the input layer specifications are the design parameters that are used in the PA configuration, and the output layer represents and predicts the outcomes, such as $G_{p}$$P_{out}$, and PAE for the inserted input data.

In this phase, the selected topology from the Phase III, which is based on the lumped elements, is used to obtain the configuration based on the transmission lines (TLs) through ’S-parameter’ simulation. Afterwards, the values of TLs are iterated randomly to obtain a suitable amount of data. Hence, by the trained DNN, the optimal design parameters that result in targeted outcomes are predicted.

## 3. Practical Execution of Various DNNs

The proposed approach was performed by arranging the execution environment with an Intel Core i7-4790 CPU @ 3.60 GHz equipped with 64.0 GB RAM, on which 2024 Keysight ADS with 2024 MATLAB tools were set. This section is devoted to presenting the practical implementations of various DNNs, including classification and regression BiLSTM-based DNNs with GANs.

The proposed method was executed with the help of a transistor model, Ampleon CLF1G0060-10, for which TCAD simulations [31] were performed for nonlinear device physical analysis. The X-parameters were characterized by the fundamental frequency $f_{0}$. Here, a set of random incident waves, $A_{11}$, $A_{12}$,..., $A_{15}$, with the reflected waves $B_{11}$, $B_{12}$,..., $B_{15}$ were used for generating the dataset in which the total data was divided with proportions of 70%, 15%, and 15% for $X_{\mathrm{Train}}$, $X_{\mathrm{Val}}$, and $X_{\mathrm{Test}}$, respectively. In total, 1500 random *A*s with *B*s (i.e., real and imaginary parts) were generated, and with this dataset (which was focused on the operational bandwidth), a BiLSTM-based DNN was trained. The ’adam optimization’ algorithm and ’standard gradient descent’ algorithm were executed for updating the weights and biases of the network.

**Figure 5:**
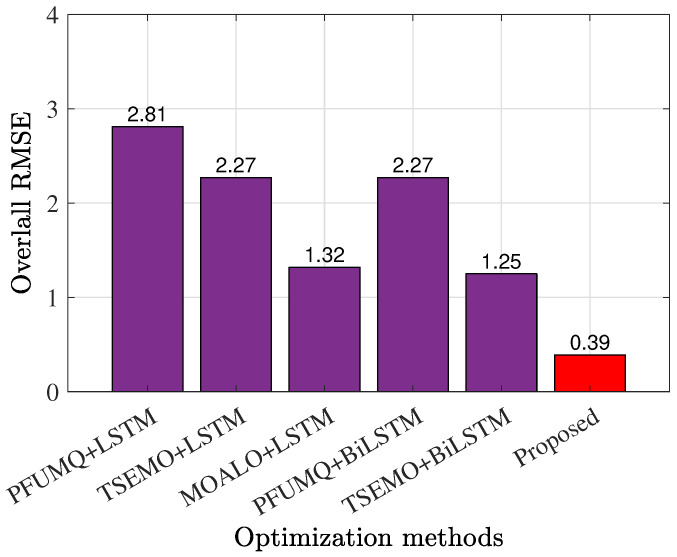
RMSE representations for various employed optimizations for the trained DNN with X-parameters in Phase I.

As we discussed in the previous section, the target of training this DNN with Xparameters was to obtain the accurate and optimal hyperparameters, including the number of hidden layers and neurons. For this case, we proposed the utilization of multi-objective optimization methods such as the Pareto front using modified quicksort (PFUMQ), Thompson sampling efficient multi-objective optimization (TSEMO), and multi-objective ant lion optimizer (MOALO) algorithms [32] which were employed with both LSTM and BiLSTM structures. As Figure 5 presents, the proposed method, that is, the utilization of the BiLSTM structure with the MOALO method, reached an overall RMSE factor of 0.39, which was more effective and accurate than other reported methods. Additionally, it was demonstrated that the BiLSTM topology was more powerful than the LSTM structure. The concluded hyperparameters that resulted in such a 0.39 RMSE factor included five hidden layers with 200 neurons in each layer. This kind of information (i.e., hyperparameters) would be used later for training DNNs of Phases III and IV.

**Figure 6:**
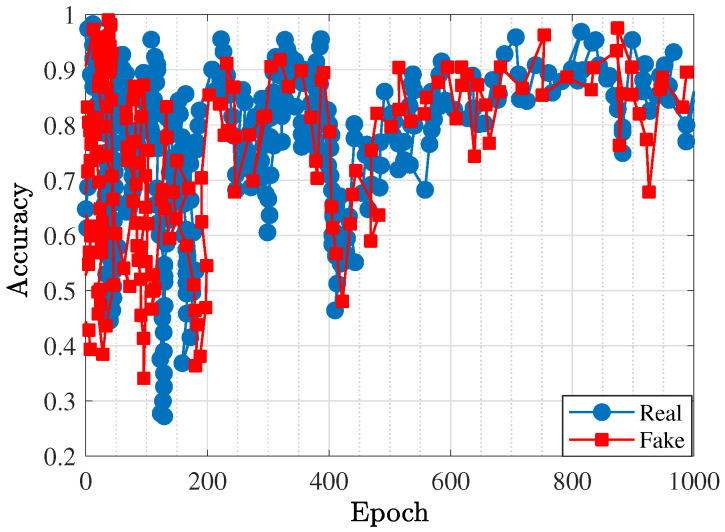
Accuracy representation for the trained GAN.

**Figure 7:**
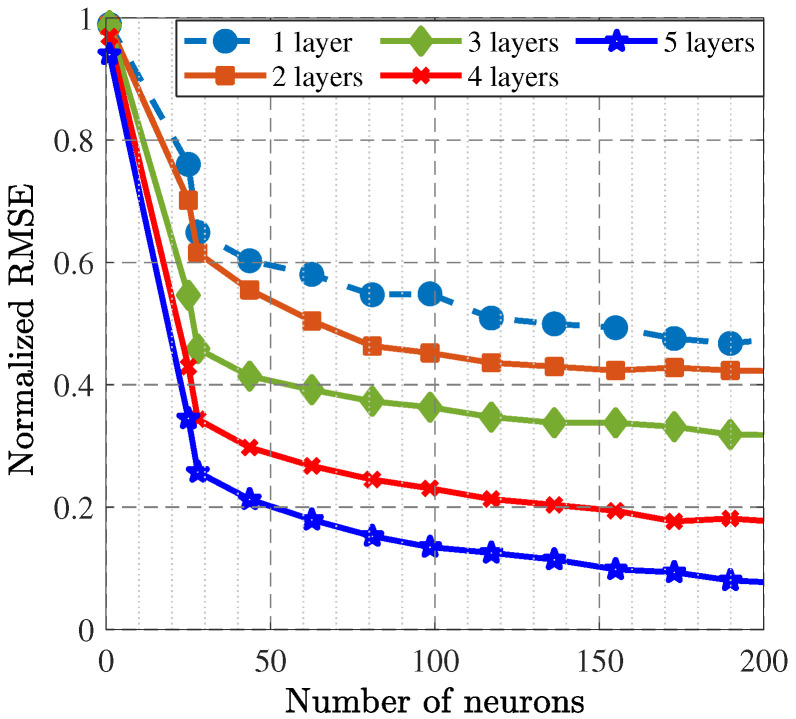
RMSE presentation for the trained classification BiLSTM-based DNN in terms of the number of hidden layers and neurons.

**Figure 8:**
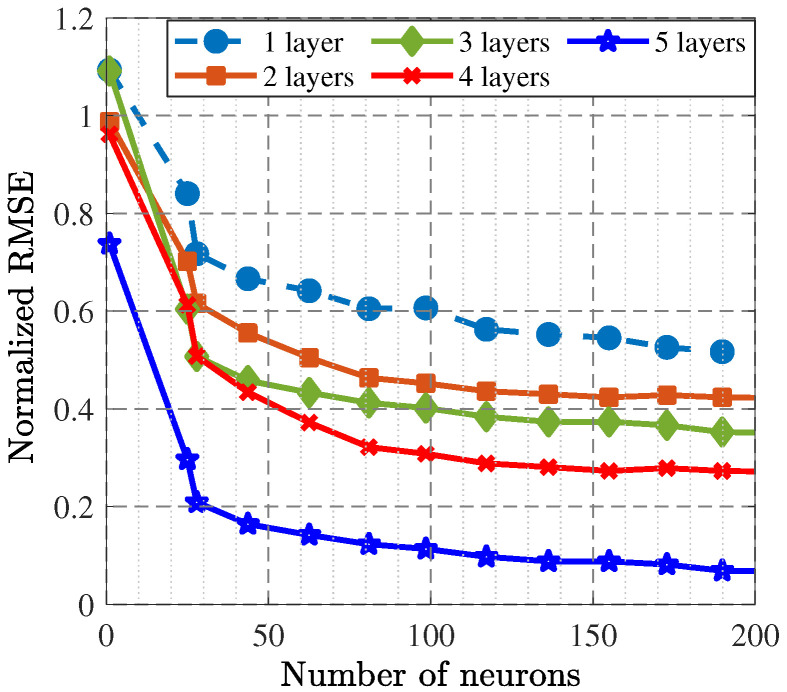
RMSE presentation for the trained regression BiLSTM-based DNN in terms of the number of hidden layers and neurons.

As the second phase, the GAN with load–pull extrapolation through the deep image completion method was constructed, leading to the prediction of the optimal gate and drain impedances that could be used for the SRFT method. The suitable size of the dataset was achieved by random iteration and collecting the load–pull simulation results. In total, 6200 data points were extracted with 32 × 32-pixel images for which 64 filters were executed. Additionally, five-by-five filters were used for the transposed convolution layers and convolution layers. For this kind of network, the MOALO method was used as well for construction. Figure 6 presents the accuracy of the trained network in terms of the RMSE, which demonstrates that in the 1000*^th^* epoch, acceptable accuracy was obtained. With the help of this trained network, the optimal gate and drain impedances were predicted, as Table 1 shows. Figure 7 and Figure 8 present the accuracy of the trained BiLSTM-based DNNs, which demonstrate that when the hyperparameters included five hidden layers with 200 neurons (similar to the one predicted in Phase I through X-parameters), the accuracy factor was less than 0.1.

**Figure 9:**
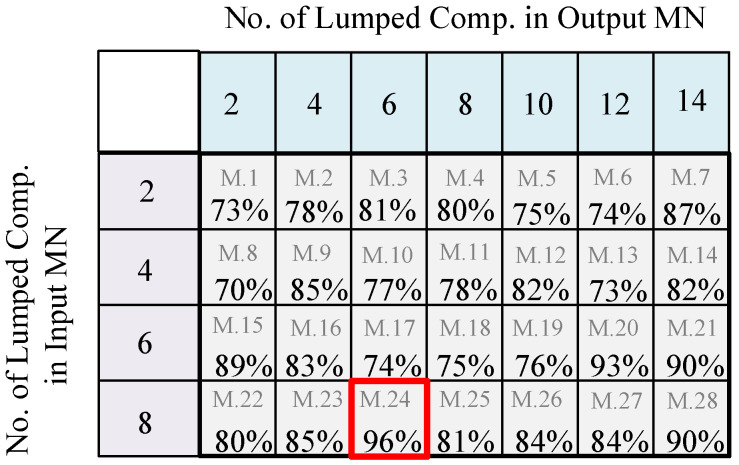
Percent predictions for various PA configurations that could be fitted to the targeted specifications.

With the help of estimated impedances, the SRFT method was employed to generate four different MNs on the input side and seven various MNs on the output side, including lumped elements (i.e., capacitors (Cs) and inductors (Ls)) in ladder formats. The classification BiLSTM-based DNN was trained with the help of 28 (i.e., 4 × 7) different generated PA configurations. As Figure 9 shows, the 24th model included eight lumped elements on the input side and six lumped elements on the output side, resulting in the most suitable topology for the employed GaN HEMT device (i.e., Ampleon CLF1G0060-10) with an accuracy of 96%. For this network, a total of 3500 data points were achieved by randomly iterating the values of lumped elements in various configurations.

**Figure 10:**
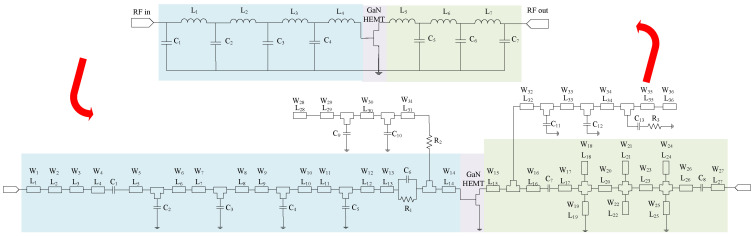
Conversion of selected lumped-element PA in to TL-based design through S-parameter simulation using keysight ADS tool.

The last DNN, a regression BiLSTM-based DNN, was constructed for obtaining the optimal geometric values. For this case, firstly, the lumped-element-based PA configuration was converted into a TL-based design with the help of ’S-parameter’ simulation, as Figure 10 shows. The PA was biased with 50 (V) and 40 (mA) and employed on Rogers RO4350B substrate with $\varepsilon_{r}$ = 3.66 and a thickness of 0.508 mm. For this network also, the design parameters of TLs, such as width (W) and length (L), were iterated randomly, resulting in 4700 data points for training the network, resulting in optimal geometric values. The values of capacitors with inductors achieved through the SRFT method, along with the TL values predicted through the DNN, are summarized in Table 2 and Table 3, respectively.

## 4. Simulation Results of Optimized PA Through Automated Proposed Methodology

With the help of the proposed method, a PA operating from 1.8 GHz to 2.2 GHz was designed and optimized with the help of the Ampleon CLF1G0060-10 GaN HEMT device. This section is devoted to presenting the simulation results for the optimized PA for which the automated approach was employed. In total, the whole optimization process lasted 5 hours and 45 minutes. Additionally, at the end of this section, Table 4 is presented for comparison with recent works.

**Figure 11:**
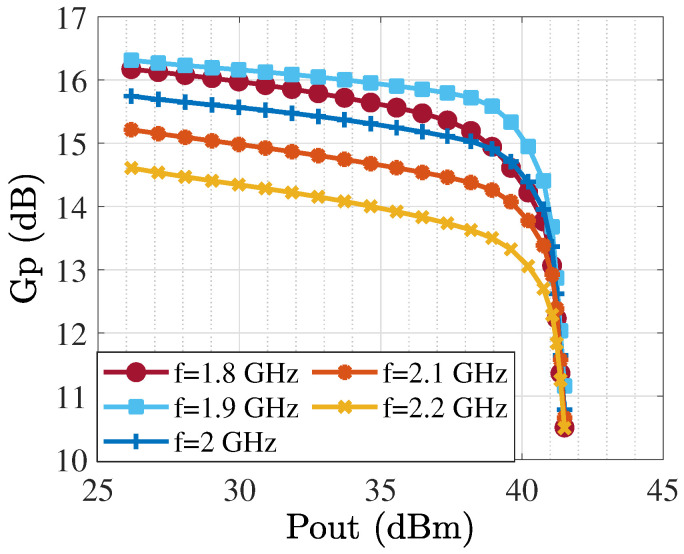
$G_{p}$ performances of optimized PA for various frequencies.

**Figure 12:**
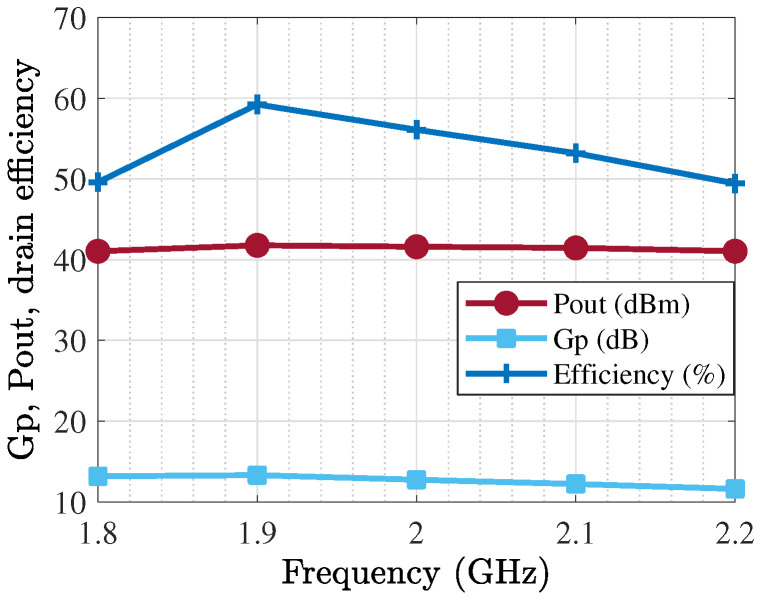
$P_{out}$, $G_{p}$, and $\eta_{D}$ performances of optimized PA over bandwidth.

**Figure 13:**
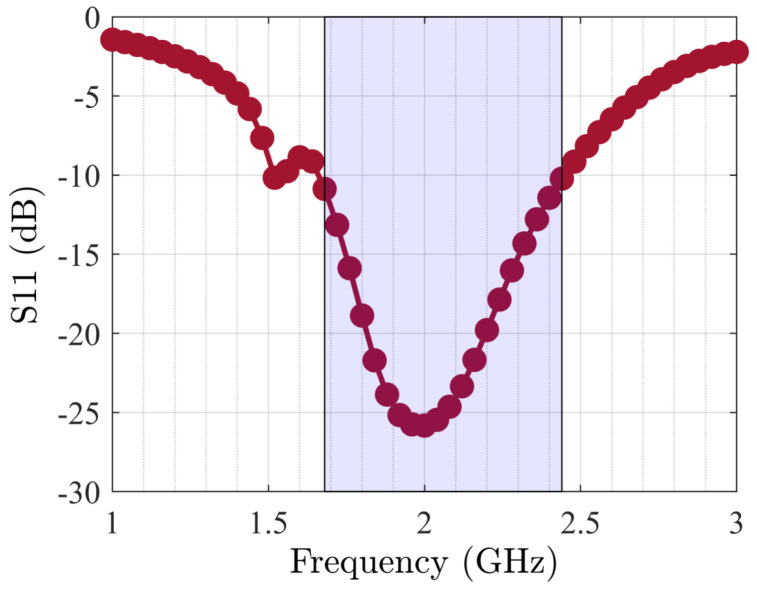
$S_{11}$ results of optimized PA over large bandwidth.

**Figure 14:**
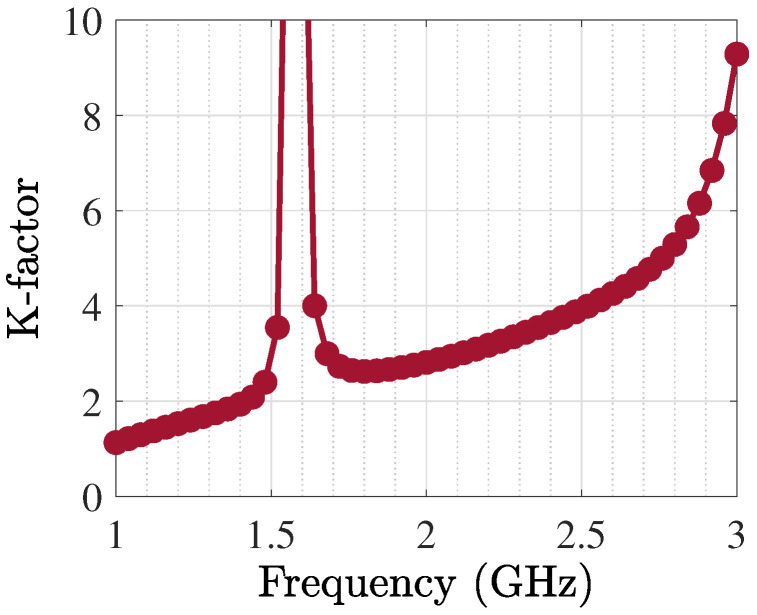
Stability factor of optimized PA.

The TL-based PA was generated and optimized, as Figure 10 shows, and related outcomes in terms of various specifications are presented from Figure 11 and Figure 12. The operational bandwidth of the optimized PA was from 1.8 GHz up to 2.2 GHz, as shown in Figure 13, and the $G_{p}$ performances of the PA over various frequencies are depicted in Figure 11. From another point of view, as Figure 12 shows, at the presented bandwidth, the $G_{p}$ specification reached around 10 dB with a drain efficiency ($\eta_{D}$) of more than 50% at 40 dBm output power. Additionally, the stability factor (i.e., K-factor) is important in designing any PA circuit; hence, Figure 14 shows a representation of this from 1 GHz to 3 GHz, which shows a greatly accepted result in the determined bandwidth.

## 5. Conclusions

In this work, we present an automated intelligence-based optimization process for predicting an optimal PA configuration along with sizing a PA operating from 1.8 GHz to 2.2 GHz. For this case, this approach is based on the implementation of BiLSTMbased DNNs along with the deep learning image completion method, using a GAN. The constructed DNN with X-parameters is the most significant phase of this overall methodology since the hyperparameters of the next DNNs are determined in this step with the help of multi-objective optimizations. Here, various optimizations are employed and compared with the LSTM topology as well, and it is observed that the BiLSTM topology with the implementation of the MOALO algorithm can have the most accurate prediction. After that, the GAN is constructed for estimating the optimal impedances that can be used in the SRFT method. Lastly, the classification with regression BiLSTM-based DNNs is employed for predicting the optimal PA configuration by sizing the MNs, respectively. The proposed approach is executed fully automatically without any human interruptions and it is flexible enough to be employed for any transistor model at any frequency range. As a result, the overall efforts for designing and optimizing high-performance PAs are reduced effectively without any manual breaks. As future work, improvements can be made by using a powerful execution environment for reducing the time consumption. Other types of DNNs, along with optimization methods, can be considered for any possible improvements.

## Figures and Tables

**Table 1:** Estimated load–pull outcomes at 3 dB gain compression with the help of the trained GAN.

Freq. (GHz)	Gate Impedance	Drain Impedance	PAE (%)	$P_{\mathrm{out}}$ (dBm)	$G_{p}$ (dB)
1.8	4.8-j5.8	20-j36	63	40	17.5
1.9	4.6-j3.9	21-j29.02	60.71	40.04	16.8
2	4.5-j3.4	21.02-j32.93	60.09	39.74	16.9
2.1	5.02-j2.5	19.81-j32.29	62.07	41	16.59
2.2	5.25-j1.8	18.23-j29.59	61.62	41.29	16.99

**Table 2:** Design parameter values for the lumped-element PA depicted in Figure 10. All capacitors (Cs) and inductors (Ls) are in pF and nH units, respectively.

$C_{1}$	3.5	$L_{1}$	1.7
$C_{2}$	25.3	$L_{2}$	0.3
$C_{3}$	143	$L_{3}$	0.15
$C_{4}$	27.7	$L_{4}$	0.3
$C_{5}$	9.3	$L_{5}$	4.3
$C_{6}$	12.7	$L_{6}$	1.3
$C_{7}$	3.15	$L_{7}$	2.2

**Table 3:** Design parameter values for optimized TL-based PA depicted in Figure 10; width (W) and length (L) of TLs are in mm, capacitors are in pF, and resistors are in Ω units.

$W_{1}$	4.8	$W_{10}$	1.5	$W_{19}$	4.1	$W_{28}$	1
$W_{2}$	4.0	$W_{11}$	1.5	$W_{20}$	3.7	$W_{29}$	1
$W_{3}$	3.0	$W_{12}$	1.5	$W_{21}$	5.3	$W_{30}$	1
$W_{4}$	1.07	$W_{13}$	3	$W_{22}$	5.3	$W_{31}$	1
$W_{5}$	1.5	$W_{14}$	1	$W_{23}$	3.7	$W_{32}$	1
$W_{6}$	1.5	$W_{15}$	4	$W_{24}$	3.7	$W_{33}$	1
$W_{7}$	1.5	$W_{16}$	3.7	$W_{25}$	8	$W_{34}$	1
$W_{8}$	1.5	$W_{17}$	3.7	$W_{26}$	1	$W_{35}$	1
$W_{9}$	1.5	$W_{18}$	4.1	$W_{27}$	1	$W_{36}$	8
$L_{1}$	1.6	$L_{10}$	1	$L_{19}$	3.6	$L_{28}$	8
$L_{2}$	1.7	$L_{11}$	1	$L_{20}$	1.6	$L_{29}$	1
$L_{3}$	1.8	$L_{12}$	1	$L_{21}$	5.5	$L_{30}$	1
$L_{4}$	3	$L_{13}$	2	$L_{22}$	6.8	$L_{31}$	26.4
$L_{5}$	1	$L_{14}$	0.2	$L_{23}$	1.6	$L_{32}$	26.4
$L_{6}$	1	$L_{15}$	0.2	$L_{24}$	3.4	$L_{33}$	1
$L_{7}$	1	$L_{16}$	8.16	$L_{25}$	1.7	$L_{34}$	1
$L_{8}$	1	$L_{17}$	1.6	$L_{26}$	1.6	$L_{35}$	1
$L_{9}$	1	$L_{18}$	5.4	$L_{27}$	1.6	$L_{36}$	8
$C_{1}$	40.9	$C_{2}$	0.28	$C_{3}$	0.04	$C_{4}$	1.08
$C_{5}$	3.6	$C_{6}$	1.6	$C_{7}$	27.50	$C_{8}$	24.5
$C_{9}$	10e3	$C_{10}$	2.2	$C_{11}$	2.2	$C_{12}$	10e3
$C_{13}$	10e6	$R_{1}$	10	$R_{2}$	10	$R_{3}$	10

**Table 4:** Summary of various methodologies employed for designing and optimizing amplifiers in recently published studies.

Ref.	Method	Goal(s) of paper
[10]	Deep learning-based CNN	- Estimating the scattering parameters of pixelated electromagnetic layouts
[33]	Deep CNN-based surrogate model	- Estimating scattering parameters
[34]	Pareto optimization	- Automatic design of low-noise amplifier with CMOS-based technology
[35]	Bayesian neural network	- Reducing the simulation consumption time for automatically designing a Doherty PA
[36]	Machine learning	- Designing a PA with 250 nm indium phosphide technology
[37]	Simulated annealing algorithm	- Presenting a design method for matching networks of PAs
[38]	Differential evolution multi-objective particle swarm optimization algorithm	- Improving the global search capability in the optimization process
This work	Co-implementation of a GAN with BiLSTM-based DNNs with multi-objective optimizations	- Obtaining the optimal hyperparameters of DNNs;
		- Predicting the optimal gate and drain impedances of the transistor; - Estimating the optimal PA structure; - Optimizing the PA’s design parameters for improving the overall specifications.

## Data Availability

No new data were created or analyzed in this study. Data sharing is not applicable to this article.
